# Bilateral Facial Palsy: A Clinical Approach

**DOI:** 10.7759/cureus.14671

**Published:** 2021-04-25

**Authors:** Alvin Yang, Vikram Dalal

**Affiliations:** 1 Family Medicine, Schulich School of Medicine & Dentistry, Western University, London, CAN

**Keywords:** bilateral, facial, palsy, paralysis, guillain-barre syndrome (gbs), sarcoidosis, pregnancy, puerperal, bell's palsy, lyme's disease

## Abstract

Bilateral facial palsy (BFP) is exceedingly rare, representing only 0.3%-2.0% of facial palsy cases. Unlike unilateral facial palsy, it is often caused by a serious underlying systemic disease and therefore warrants urgent medical intervention. The differential diagnosis is broad, and detailed history, physical examination, and investigations are essential for identifying the etiology. Common acquired causes in existing case series include Lyme disease, Guillain-Barré syndrome, sarcoidosis, trauma, and Bell’s palsy. Palsy that develops rapidly is often caused by trauma, infections, or autoimmune disorders, whereas slow progressive palsy suggests neoplastic diseases. While management varies by etiology, the physician can consider early empiric corticosteroids given their efficacy in numerous differential diagnoses. Antivirals can be considered in those with a strong history of viral prodrome. In this paper, we present the case of a puerperal patient with BFP and discuss its differential diagnosis, diagnostic approach, and management.

## Introduction

Unilateral facial palsy is relatively common with an incidence of 20-25 per 100,000 population, where the majority of cases (>50%) are attributed to Bell’s (i.e. idiopathic) palsy (BP) [1,2]. In comparison, bilateral facial palsy (BFP) is exceedingly rare, representing only 0.3%-2.0% of facial palsy cases [3,4]. BFP is often a finding of a serious systemic disease, with BP seldom being the cause (<20%) [2,3,5]. Many of these diseases are potentially life-threatening and therefore warrant urgent medical intervention [1]. In this paper, we present the case of a puerperal patient with BFP and discuss its differential diagnosis, diagnostic approach, and management.

## Case presentation

A 31-year-old Caucasian female presented to the emergency department (ED) with BFP. She had delivered her daughter seven days prior and noticed hypogeusia the same day. She subsequently developed left-sided facial weakness on day 4 and right-sided facial weakness on day 7. She denied diplopia, facial numbness, otological symptoms, dysphagia, as well as jaw and neck weakness. She had no sensory changes, pain, or weakness in the body. There were no bladder or bowel symptoms, nor head or neck trauma. She had no recent infectious symptoms, sick contacts, or travel. There was no history of facial palsy. Of note, she had received a rubella vaccine two days after the delivery of her child.

On examination, the patient had stable vital signs and was in no apparent distress. Her facial strength was decreased bilaterally with an inability to elevate eyebrows, puff out cheeks, or smile. Two millimeters of inferior scleral margins remained visible bilaterally, indicating inadequate closure of both eyelids. There was no fatiguability. The remainder of the cranial nerve examinations were normal. She had intact sensation, strength, tone, and reflexes in bilateral upper and lower extremities. Gait was normal and plantar response was down-going. There were no rashes.

Initial blood work demonstrated complete blood count (CBC) to be within normal range. Similarly, serum glucose, sodium, potassium, chloride, bicarbonate, magnesium, calcium, and phosphate were also within normal range. C-reactive protein (CRP) was very minimally elevated at 15.2mg/L.

Our patient was referred to and assessed by the Urgent Neurology Clinic within hours of presenting to the ED. Interestingly, further investigations were not performed, possibly due to her relatively benign history and exam, lack of red flags, and puerperal association. She was clinically diagnosed with bilateral BP and discharged home with eye patching only. Follow-up at eight weeks later revealed a complete resolution of the BFP without any additional symptoms. Therefore, a final diagnosis of bilateral BP in puerperium was established and the patient was discharged without further investigations or follow-up. While a more extensive workup was not performed, this case nonetheless promotes discussion on additional management considerations.

## Discussion

Differential diagnosis

Tables 1, 2 show compiled etiologies from previous literature, including BFP during puerperium [1,2,6-9].

**Table 1:** Infectious etiologies of bilateral facial palsy CMV, cytomegalovirus; EBV, Epstein-Barr virus; GBS, Guillain-Barré syndrome; HIV, human immunodeficiency virus; HTLV-1, human T-lymphotropic virus; HZV, herpes zoster virus.

Viral	Bacterial	Spirochete, protozoa, fungi
Arbovirus	Diphtheria	Borreliosis (Lyme disease)
Brainstem encephalitis	Intracranial abscess (mycoplasma pneumoniae)	Leptospirosis
Coxsakie virus	Leprosy	Malaria
CMV	Meningitis	Syphilis
EBV	Otitis media (bilateral)	Cryptococcal meningitis
GBS (Miller Fisher syndrome)	Scarlet fever	
HIV	Tetanus	
HTLV-1	Tuberculosis	
HZV (Ramsay Hunt syndrome)		
Influenza		
Mumps		
Poliomyelitis		

**Table 2:** Non-infectious etiologies of bilateral facial palsy SJS, Stevens-Johnson syndrome; SLE, Systemic lupus erythematosus.

Autoimmune	Congenital	Metabolic	Neoplastic	Neurological/Neuromuscular	Traumatic	Vascular	Iatrogenic	Idiopathic
Amyloidosis	Facial musculature absence	Acute porphyria	Acoustic neuroma	Benign intracranial hypertension	Facial injury/laceration	Brainstem cavernous hemangioma	Arterial embolization	Bell’s palsy
Sarcoidosis	Myotonic dystrophy	Diabetes mellitus	Cholesteatoma	Vestibular schwannomas	Forceps birth trauma	Polyarteritis nodosa	Rabies antiserum inoculation	
SJS	Melkersson-Rosenthal syndrome	Wernicke-Korsakoff syndrome	Ependymoma	Bulbospinal neuropathy	Parotid surgery	Pregnancy	Vaccine	
SLE	Moebius syndrome		Leukemia	Multiple sclerosis	Skull base fracture	Puerperium		
	Sclerosteosis		Lymphoma	Myasthenia gravis		Temporal arteritis		
	Thalidomide embryopathy		Pontine glioma	Parkinson’s disease				
			Von Recklinghausen’s disease	Pseudobulbar/bulbar palsy				
				Pilocytic astrocytoma				

The most common acquired etiologies reported across case series were Lyme disease, Guillain-Barré syndrome (GBS), sarcoidosis, trauma, and BP [1,6,9,10]. Palsy that develops rapidly is often caused by trauma, infections, or autoimmune disorders. Slow progressive palsy suggests neoplastic diseases. Synchronous palsy with involvement of the contralateral side within 30 days may represent systemic diseases whereas synchronous palsy with involvement of the contralateral side after 30 days may represent more localized inflammatory conditions [6].

Diagnostic approach

Detailed history, physical examinations, and investigations are essential for identifying the etiology. History should include symptom characterization (e.g. time sequence of onset, synchronicity, taste change), associated symptoms (e.g. diplopia, facial numbness, otological symptoms, aphasia, vesicles), relevant risk factors (e.g. viral prodrome, camping, immunizations), and prior palsy history [2]. The physical examination should assess all systems, paying special attention to localizing neurological deficits. Classifications include unilateral vs. bilateral distribution, central vs. peripheral neuropathy, upper motor neuron vs. lower motor neuron lesion, myotome and dermatome distribution, proximal vs. distal muscle weakness, and fatiguability. Clinical syndromes can raise a high index of suspicion for specific diseases. Headache, visual disturbance, and jaw claudication suggest temporal arteritis, while headache and papilledema suggest benign intracranial hypertension. Intermittent monosymptomatic attacks compatible with demyelination (e.g. optic neuritis, painless binocular diplopia, cerebellar syndrome) may indicate multiple sclerosis. Myasthenia gravis is characterized by fluctuating skeletal muscle weakness and fatiguability, often with ocular involvement (e.g. ptosis, diplopia). Bradykinesia, resting tremor, rigidity, and postural instability are hallmarks of Parkinson's Disease. Encephalopathy, oculomotor dysfunction, and gait ataxia form the classic triad for Wernicke-Korsakoff syndrome.

Initial bloodwork should include a CBC, electrolytes, creatinine, and blood glucose. Additional workup should be guided by history and physical examination. Investigations for the patient with infectious symptoms may include a chest X-ray, heterophil count, fluorescent treponemal antibody test, Lyme titer, and human immunodeficiency virus screen. Focal neurological deficits (e.g. asymmetric sensorimotor hearing loss) should be followed with neuroradiology (i.e. computed tomography, magnetic response imaging) to explore intracranial pathology such as acoustic neuroma, ependymoma, pilocytic astrocytoma, and brainstem cavernous hemangioma. Non-specific systemic symptoms (e.g. fatigue, arthralgia, fever) may prompt inflammatory and autoimmune biomarkers (e.g. CRP, erythrocyte sedimentation rate, antinuclear antibodies, antineutrophil cytoplasmic antibodies, antiphospholipid antibodies) for conditions such as systemic lupus erythematosus, amyloidosis, and polyarteritis nodosa. Blood smear and lymph node biopsy should be considered in the patient with malaise and lymphadenopathy to investigate leukemia and lymphoma. Other adjuncts include, but are not limited to, lumbar puncture for benign intracranial hypertension and tissue biopsy for amyloidosis. Urgent Neurology consult should be initiated, especially as all these investigations may not be available in an office or ED setting. The patient without an obvious etiology should be hospitalized to rule out life-threatening diseases. Only after excluding these serious possibilities should a diagnosis of BP then be considered [2,6,7].

Management

Specific management of the diverse differential diagnoses is beyond the scope of this paper; rather, we will briefly review BFP in pregnancy and puerperium as well as select systemic diseases with increased prevalence.

There is a higher incidence of facial palsy in pregnant (45/100,000 births) vs. non-pregnant (17/100,000 births) women. It presents almost exclusively in the third trimester or postpartum period [11]. Although there are case reports of BFP in pregnancy, the incidence is unknown [12,13]. The pathophysiology is poorly understood, but proposed etiologies include cortisol-mediated immunosuppression leading to viral reactivation, fluid retention within the fallopian canal causing compressive neuropathy, a hypercoagulable state producing microthrombi or emboli that occlude vasa nervorum, hormone level changes, and hypertension [11,14,15]. Diagnosis involves excluding other contributory systemic diseases. Prednisone 25mg twice daily for 10 days, started within three days of symptom onset, is usually associated with full recovery. This is generally safe for the fetus during pregnancy and neonate during breastfeeding. Concurrent valacyclovir 1000mg three times daily for seven days can be considered but has less supporting evidence. Delays in management are associated with worse outcomes, including persistent palsy [14]. Neonatal outcomes were favorable regardless of treatment received [11].

Lyme disease, caused by the tick-borne Borreliella infection, is the most common infectious cause of BFP (30%-35% of cases) [16]. In the early localized phase, it resembles a viral syndrome and commonly produces regional lymphadenopathy as well as erythema migrans (“bull’s eye rash”). Early dissemination and late disease manifest in multiple systems, including diagnoses such as carditis, arthritis, and encephalopathy. The clinical features of each stage can overlap, and patients can present in a later stage without symptoms suggestive of earlier disease. Neuroborreliosis presents in early dissemination, with facial paralysis being the most common manifestation (11% of cases, of which 30%-40% are bilateral) [6,10]. Other neurologic manifestations include lymphocytic meningitis, radiculopathy, and peripheral neuropathy. Diagnosis is supported by a positive serum antibody titer to Borrelia IgG. Doxycycline 100mg twice daily for 14 days is the treatment of choice and should be started immediately without waiting for serological confirmation. The treatment duration can be extended to up to 28 days in late disease (e.g. presence of carditis or arthritis). The prognosis is generally excellent with appropriate antibiotic therapy.

GBS is an acute immune-mediated polyradiculoneuropathy caused by cross-reaction to a preceding infection. Cardinal features include rapidly progressing symmetric muscle weakness accompanied by depressed deep tendon reflexes. GBS typically involves legs, arms, trunk, and face, with severity ranging from mild ambulation difficulty to nearly complete paralysis of all extremity, respiratory, bulbar, and facial muscles. Cranial nerves VII, IX, and X are most commonly affected in cranial neuropathy [1,2]. The facial nerve is involved in 27%-50% of cases and BFP is seen in 50% of these patients [2]. Although BFP can be the first and only symptom of GBS, it is a poor prognostic sign (occurs in up to 50% of fatal cases) as it represents high motor involvement and suggests possible respiratory failure [7,16]. The initial diagnosis is based on clinical presentation. Subsequent diagnostic certainty is supported by positive cerebrospinal fluid (elevated protein with normal white blood cell count) analysis and abnormal nerve conduction studies. Supportive care is the mainstay of treatment since up to 30% of cases develop respiratory failure requiring mechanical ventilation [17]. While 95% of cases demonstrate spontaneous remission and most patients show symptom improvement within four weeks, plasma exchange and intravenous immune globulin may be used within 10 days of symptom onset to accelerate recovery [2,6,7].

Sarcoidosis is a multisystem granulomatous disorder of unknown etiology characterized by noncaseating granulomas. The pulmonary system is commonly affected (e.g. bilateral hilar adenopathy, pulmonary reticular opacities), although many patients have extrapulmonary sarcoid (e.g. eye and skin lesions) symptoms. Neurosarcoidosis usually manifests as cranial neuropathies, with the facial nerve being most commonly affected (unilateral palsy in 1%-3% and bilateral palsy in 0.1%-0.2% of patients) [10]. Other neurologic manifestations include neuroendocrine dysfunction, encephalopathy, and myelopathy. Diagnosis is supported by elevated serum angiotensin converting enzyme (ACE), tissue biopsy of affected organs, and enlarged lymph nodes on chest CT. Glucocorticoids for 4-6 weeks is used to reduce symptoms, although its effect on the natural course of neurosarcoidosis is unknown [2]. Patients with facial palsy are often treated with prednisone 0.5mg/kg/day for 2 weeks and improve over 2-4 weeks [18].

BP is defined as acute facial palsy of unknown cause, with postulated mechanisms involving hereditary, vascular, infective, and inflammatory autoimmune factors [10]. It is often preceded by a viral infection (60% of cases), with associated features including face or ear pain (50%), facial numbness (40%), dysgeusia (50%), and tongue numbness (20%) [1]. It is diagnosed clinically and treated with glucocorticoids (i.e. prednisone 60-80mg/day for seven days). Antivirals (e.g. valacyclovir 1000mg three times daily for 7-10 days) can be used in cases of severe facial palsy, especially in Ramsay-Hunt Syndrome. One side of the face often recovers immediately with the opposite side taking weeks to several months to fully recuperate [8,10].

While BFP management varies drastically by etiology, the physician can consider early empiric corticosteroids given their efficacy in numerous differential diagnoses (e.g. pregnancy, sarcoidosis, systemic lupus erythematosus, neoplastic lesions, multiple sclerosis, temporal arteritis, BP), provided there are no contraindications. Antivirals or antibiotics (e.g. doxycycline) can be considered in those with a strong history of viral prodrome or bacterial infection (e.g. Lyme disease), respectively [9]. Although empiric management may be similar to that of unilateral facial palsy, patients must be followed closely to adapt therapy to new clinical, laboratory, and imaging findings. While the prognosis of BFP depends on the etiology, bilateral BP seems to recover as well as the unilateral form [1].

## Conclusions

BFP is a rare presentation caused by a myriad of disparate conditions, many of which may be life-threatening and warrant immediate intervention. Common systemic diseases include Lyme disease, GBS, and sarcoidosis. It is prudent to have a diagnostic approach and consult Neurology urgently. Early empiric management with corticosteroids, antivirals, or antibiotics may be considered based on initial clinical and laboratory assessment.
